# Attitudes toward finitude in the cinema of Federico Fellini: Light on older adults

**DOI:** 10.3389/fpsyg.2022.974012

**Published:** 2022-07-29

**Authors:** Hélio José Coelho-Junior, Emanuele Marzetti

**Affiliations:** ^1^Department of Geriatrics and Orthopedics, Università Cattolica del Sacro Cuore, Rome, Italy; ^2^Fondazione Policlinico Universitario “Agostino Gemelli” IRCCS, Rome, Italy

**Keywords:** ageism, fear of death, depression, suicide, elderly

## Introduction

Thoughts about finitude are common in the life of older adults owing to the numerous events that surround this period, including the increased prevalence of diseases (Quiñones et al., 2016), the decline in physical and cognitive functions (Marzetti et al., 2018; Coelho-Junior et al., 2021), the constant contact with ageist representations (Levy et al., 2022), and the death of close people (Ng et al., 2021). Failure to adequately cope with these factors may render those thoughts frequent and pervasive (Ng et al., 2021; Rababa et al., 2021). Important psychosocial theories have explored the matter and psychological circuits have been proposed whereby attitudes toward death are created, feedback is maintained, and implications for people's life ensue (Erikson, 1998; Frankl, 2006).

Despite the importance of the subject, little attention has been paid as to how finitude can be discussed and explored with older adults. Cinematherapy is a modern therapeutic technique in which the therapist selects a movie to explore aspects of life and evoke mental processes to stimulate self-discovery, provide strategies to manage hardship, and offer psychological support (Schulenberg, 2003; Dumtrache, 2014; Hankir et al., 2015; Correia and Barbosa, 2018; Smieszek, 2019). The type of movie is selected according to the aspect targeted by the therapist (Smieszek, 2019). In general, popcorn cinema is expected to contribute to emotional release, evocative cinema to help people learn about themselves, and cathartic cinema for opening different levels of emotions and psyche (Smieszek, 2019). In addition, people might identify themselves with some characters and use them as role models (Smieszek, 2019). Specifically, the attitude and behavior of specific characters through the narratives might stimulate people to reflect on their lives and, with appropriate support, restructure their universe (Schulenberg, 2003; Dumtrache, 2014; Hankir et al., 2015; Correia and Barbosa, 2018; Smieszek, 2019).

Federico Fellini is one of the most famous screenwriters and film directors of all time (Bondanella, 2002). The cinema of Fellini represents a complex, elegant, and sometimes contradictory combination of poetic, lyrical, and visual images (Bondanella, 2002). He is frequently compared to or even recognized as a master of the Italian Renaissance owing to his ability to manage light and sketch out every detail (Bondanella, 2002). Another aesthetic characteristic of his cinema is the obsession to “enter” in the human brain and explore the imagination, the irrational, and the subconscious (Bondanella, 2002). As such, Fellini's movies might represent a valuable material to reflect on some paradigms of human existence. His old people characters found it difficult to understand and realize what they have achieved during life. This scenario might provide the bases for the development of numerous negative feelings, including suffering, despair, and psychological distress (e.g., depressive symptoms, feeling of failure, anxiety).

These premises led us to explore facets that might represent a trigger for negative events in older adults in one of the most famous Fellini's movies, La dolce vita (1960, Riama/Pathé Consortium Cinema). We based our interpretation on logotherapy and the theory of stages of psychosocial development.

## La dolce vita, 1960

The character of the study is Marcello Rubini, interpreted by Marcello Mastroianni, a Roman journalist specialized in celebrities. He relates cotemporally with three women. One of them is Emma (Yvonne Furneaux), a melodramatic person who tries to get his attention and loyalty, despite his visible disdain for her needs. Their relationship involves verbal and physical aggression, in addition to alcohol abuse and a suicidal attempt. Marcello has also issues with his father, who has problems with alcohol and extramarital affairs. They have a distant relationship. Marcello's behavior is easily comparable to his father.

Marcello is unsatisfied with his life. He frequently wonders about the importance and significance of his job and lifestyle. A good illustration of this scenario is when Marcello meets his friend, Enrico Steiner (Alain Cuny), for dinner. Many artists, poets, painters, musicians, and writers attend the meeting, and Marcello shows interest in everything that is happening that night. He is different from the man we used to see working with the paparazzi. At a certain time of the night, a woman addresses him and says:

—Steiner ha detto che tu hai due amori. E che non sai scegliere tra i due: giornalismo e letteratura. Attento alla prigione! Stai libero, disponibile… Non sposare niente (Steiner said that you have two loves and that you don't know which one to choose: journalism and literature. Watch out for prisons! Be free, available… Do not marry anything).

Marcello replies:

—Qualche anno fa ho letto le sue poesie, quando pensavo di scriverne pure io. Mi piacciono, sono forti (A few years ago I read your poetry, when I thought I would write it too. I like it, it's strong).

Then, completes his narrative letting out the truth:

—Questa è proprio l'arte che preferisco, quella che penso servirà domani: un'arte chiara… senza retorica, che non dica bugie, che non sia adulatrice. Adesso faccio un mestiere che non mi piace, ma penso spesso a quello che occorrerà domani (This is my favorite art, what I think will be needed tomorrow: a clear art…without rhetoric, that doesn't. lie, that isn't flattering. Now I have a job that I don't like, but I often think about what will be needed tomorrow).

Marcello is amazed by the atmosphere. In a moment of privacy with Steiner, he claims:

—Fammi venire più spesso qui da te… Dovrei cambiare ambiente. Dovrei cambiare tante cose. La tua casa è un vero rifugio… Io sto perdendo i miei giorni... Una volta avevo delle ambizioni, ma forse sto perdendo tutto, dimenticando tutto (Let me come here more often... I should change environment. I should change many things. Your house is a real refuge... I am wasting my days... Once I had ambitions, but maybe I'm losing everything, forgetting everything).

Steiner says that he could help Marcello change his environment, introducing him and his work to a publisher, allowing him to spend his time working on what is truly interesting to him.

The time passes and Marcello does not change his life habits. Steiner dies by suicide. And it is possible that such an end, for a figure that Marcello admired as a sage, might have influenced his life.

The film skips ahead a few years for a moment when Marcello has white hair. Marcello now is an advertising agent and, according to him, with great satisfaction. He is at a party and some people treat him with contempt due to his professional occupation. They call him: The intellectual, in a derogatory way. Marcello is visibly frustrated with his life and with the way people treat him. At the same time, it looks like it is all that he has left. He is apparently single.

## Discussion

Marcello Rubini is an interesting character who has a stable financial life but is not satisfied with his work. He does not feel that he is leaving the life he should live. His environment is surrounded by promiscuity and alcohol abuse, whereas a part of him is in love with the artistic life. He wants to do something else, such as writing literature and maybe poetry. Although a friend offers help to him change his life, he decided to stay in the advertising business. His old age is characterized by a rich lifestyle, and he continues to live in the same environment where everybody seems to be different from him. However, now he is a laughingstock and there is no apparent alternative way forward.

We propose two interpretations of this character. The first is inspired by Dr. Viktor E. Frankl's theory, logotherapy (Frankl, 2006). This approach is based on finding meaning in life and involves the ability people have to take charge of their own life (Frankl, 2006). Logotherapy understands that a problem is an opportunity for knowledge and growth and that the attitude facing this problem is what people should be concerned about (Frankl, 2006). A frequent idea is that there is always an alternative (Frankl, 2006). Lack understanding the meaning of life may result in emotional distress (Frankl, 2006).

It is not possible to conclude whether Marcello had a clear meaning in life, but the movie shows that he was clearly disturbed by the way his life was going. He had no plans for his future, although he had a clear desire to live another life. This conflict might explain his lifestyle. Indeed, people with meaning in life drink less alcohol (Copeland et al., 2020) and are more likely to be part of support groups for Sexaholics (Wnuk and Charzyńska, 2022). Instead, adults who still look for meaning in life often shown unhealthy drinking behavior (Copeland et al., 2020). Nevertheless, the impact of environmental factors and social costumes should not be disregarded.

The future of Marcello is unpredictable, also because most of his old life was not explored in the movie. However, we noted that he did not change his life habits or his field of work. Having meaning in life is positively associated with good interpersonal relationships (Mulahalilović et al., 2021). This might mean support in difficult times, which does not seem to be the case in Marcello's history. The biggest concern regarding the lack of meaning in life is the presence of depressive symptoms (Szcześniak et al., 2022) and the close association with suicidal ideation observed in older adults (Lutzman and Sommerfeld, 2022).

Another interpretation is based on Prof. Erik Erikson's stages of psychosocial development theory (Erikson, 1998). According to him, old age is a challenging period of life in which people face the finitude and examine the past in order to recognize their achievements (Erikson, 1998). A positive look at this evaluation, which recognizes mistakes as part of life, failures as an opportunity to learn, and might, in spite of everything, observe a personal growth, provides resources to live the end of life with wisdom and tranquility in relation to death (Erikson, 1998). Otherwise, old age would be lived with despair (Erikson, 1998).

Our biggest concern with Marcello is related to what and how he would see his past. A probable issue would be his decision to not even try to be a writer. It might be a trigger to an end of life of frustrations and negative thoughts, such, for instance, that his life could have been different if he had tried other roads. This is a delicate moment because death approaches, not much has been done, and despair is certainly an outcome. This scenario is commonly associated with fear of death, which evokes anxiety, depressive symptoms, and despair (Tsai et al., 2005; Tomer et al., 2013; Willis et al., 2019; Aisenberg-Shafran et al., 2021), thereby generating a self-reinforcing loop. In the end, Marcello would probably search for symbolisms that could provide him with an idea of eternity (Becker, 2011).

Another possibility is that Marcello would seek social support from close friends and family members to find meaning in life. These premises are based on the *Theory of Socioemotional Selectivity* (Carstensen et al., 1999), which states that the perception that time is passing changes the selection of life goals. When people are aware that their time is limited, their goals are expected to be based on the present, rather than creating plans for the future, and mainly involve emotional satisfaction. Such an approach offers older people support in difficult moments and serves as a source of positive emotions.

We believe that this description can be useful to therapists and health professionals who work with cinematherapy. Many old patients, mainly men, have a history of alcohol abuse and consequently have numerous regrets about mistakes of the past. Marcello seems to be an interesting character that might provide a role model for people who are entering old age. Furthermore, *La dolce vita* is a masterpiece full of art that can provide a lighter approach to the problem than other movies.

## Author contributions

Both authors listed have made a substantial, direct, and intellectual contribution to the work and approved it for publication.

## Funding

This work was supported by an Intramural Research Grant from the Università Cattolica del Sacro Cuore (D1.2020) and the nonprofit research foundation Centro Studi Achille e Linda Lorenzon. The APC was funded by the Ministero della Salute – Ricerca Corrente 2022.

## Conflict of interest

The authors declare that the research was conducted in the absence of any commercial or financial relationships that could be construed as a potential conflict of interest.

## Publisher's note

All claims expressed in this article are solely those of the authors and do not necessarily represent those of their affiliated organizations, or those of the publisher, the editors and the reviewers. Any product that may be evaluated in this article, or claim that may be made by its manufacturer, is not guaranteed or endorsed by the publisher.
